# Diagnostic Value of Virtual Touch Tissue Quantification for Breast Lesions with Different Size

**DOI:** 10.1155/2014/142504

**Published:** 2014-04-02

**Authors:** Minghua Yao, Jian Wu, Liling Zou, Guang Xu, Juan Xie, Rong Wu, Huixiong Xu

**Affiliations:** ^1^Department of Ultrasound in Medicine, Shanghai Tenth People's Hospital, Tongji University School of Medicine, No. 301, Yanchangzhong Road, Zhabei, Shanghai 200072, China; ^2^Department of Health Statistics, Tongji University School of Medicine, Shanghai 200072, China

## Abstract

*Purpose*. To evaluate diagnostic value of the virtual touch tissue quantification (VTTQ) for breast lesions with different sizes. *Materials and Methods*. Patients with 206 breast lesions were categorized into three groups according to lesion size (<10 mm, 10–20 mm, and >20 mm). Breast lesions were examined by conventional ultrasound and VTTQ, and shear wave velocity (SWV) of each lesion and adjacent normal breast tissue were measured. Diagnoses were confirmed by pathological examination after surgery. The receiver-operating characteristic curve (ROC) analyses were performed to evaluate the diagnostic value of SWV, and the area under curves (AUC) was compared among groups. *Results*. SWV of malignant lesions was much higher than that of benign lesions, whereas the difference was not obvious for lesions <10 mm (*P* = 0.15). There was statistical significant difference of AUC between lesions <10 mm and 10–20 mm (*P* < 0.05), as well as lesions <10 mm and >20 mm (*P* < 0.05). The sensitivity of lesions <10 mm was 33.33%, which was relatively low compared to other groups. * Conclusion*. According to our results, VTTQ is a promising method for breast lesions >10 mm, and further studies were warranted to improve sensitivity of VTTQ for breast lesions <10 mm.

## 1. Introduction

Breast cancer is one of the major diseases threatening women's health, and early detection and diagnosis are particularly important to reduce its mortality and prognosis improvement. Ultrasound is one of the most widely used methods to diagnose breast lesion in clinical practice. Based on the morphological features of the breast lesion, conventional ultrasound could give a preliminary diagnosis. But there is a considerable overlap between the benign and malignant lesions, which is difficult to give a specific qualitative diagnosis [1]. Acoustic Radiation Force Impulse (ARFI) is a novel elastography technique which is based on the assessment of elastic properties by acoustic pulse [2, 3]. When the ARFI is initiated, the probe emitted a short-duration acoustic pulse which caused slight vibration both in longitudinal and transverse direction. The displacement amplitudes caused by the acoustic push are reflected as elastographic image, which is the principle of VTTI (Virtual Touch Tissue Imaging). The same acoustic push which caused the lateral displacements to the push to examine how fast the resulting shear wave propagates is the foundation of VTTQ (Virtual Touch tissue Quantification; Siemens Medical Solutions, Mountain View, CA). The time to peak displacement at each lateral location is defined as shear wave velocity (SWV, m/s), which is the quantitative form of VTTQ [4]. SWV of soft tissue is slower than that of hard tissue, which provided an objective indicator of the tissue stiffness [5]. According to the previous reports, VTTQ has been used for diagnosis in organs such as liver, thyroid, prostate, pancreas, and breast [4, 6–10]. Considering the benign and malignant breast lesions may vary in their tissue stiffness, but little is known about whether the size of the lesion will make influence on the diagnosis, and we therefore aimed to investigate the diagnostic value of SWV in assessment of breast lesions, especially in different lesion size.

## 2. Materials and Methods

### 2.1. Study Design and Patients Involved

**Figure 1:**
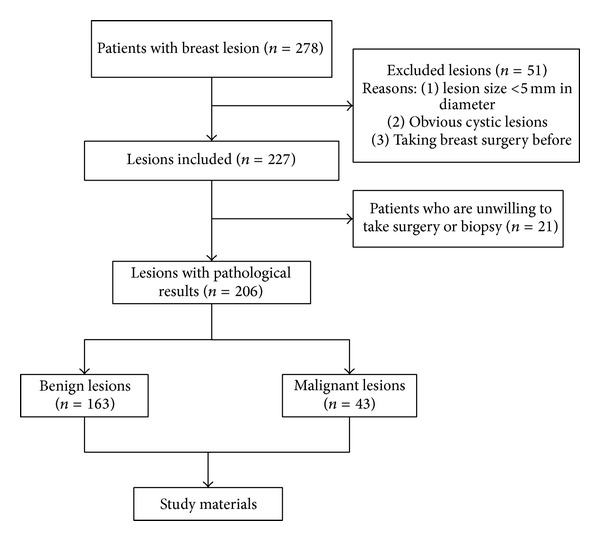
The flowchart of study design.

From July, 2011, to December, 2012, 237 patients with screen detected abnormalities or with symptoms took ultrasound examination in our hospital. After exclusion, totally 146 patients with 206 breast lesion were enrolled in our study (Figure 1). Patients excluded in the study were due to one or more of the following reasons: having (1) the maximum diameter of lesion <5 mm, (2) obvious cystic lesions, (3) taken breast surgery before, and (4) been unwilling or unsuitable to take surgery to get pathological result. All the sonographic examinations including conventional ultrasound and VTTQ were performed by one of two sonographers, who have over 10-year experience of breast ultrasonography and VTTQ detection for at least 6 months. To evaluate the interobserver reproducibility of VTTQ, another group of 30 patients was selected regardless of age, conventional ultrasound features, or lesion size and underwent the examination of VTTQ by both sonographers independently. This study was performed in strict accordance with the ethical guidelines of the Helsinki Declaration. All involved patients provided verbal informed consent to participate in this study. The Ethics Committee of the Tenth People's Hospital of Tongji University approved the consent procedures.

### 2.2. Ultrasonography Device and Measurements

**Figure 2:**
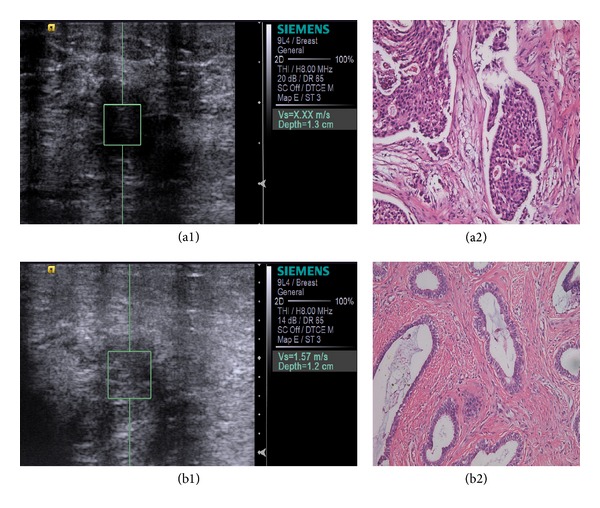
(a1) and (b1) showed the location of the region of interest (ROI). The value of SWV was automatically calculated. The pathological results of the lesions (200x) were invasive ductal carcinoma (a2) and fibroadenoma (b2), respectively.

Acuson S2000 ultrasound system (Siemens Medical Solutions, Germany) equipped with a linear 4–9 MHz multifrequency probe was used to perform both conventional B-mode ultrasound and VTTQ. After being informed with examination details, the patients were asked to take the supine position with the breast fully exposed. The probe was gently put on the breast with light pressure. The conventional ultrasound scanning was performed first and the size, boundary, echogenicity, and calcification of lesions were carefully recorded. Then the ultrasonography was adjusted to ensure the lesion was in half to one-third of the real-time image, and the boundary between lesion and adjacent normal breast tissue was clearly showed. The region of interest (ROI) was put in the center of lesion, and calcification in the ROI was avoided (Figure 2). Patients with respiratory disease or large respiratory amplitude were asked to hold breath. SWV was automatically measured by the inbuilt software and repeated for seven times. The adjacent normal breast tissue at the same depth in glandular layer was measured with the same protocol. In order to exclude the deviation, the highest and lowest measurements were removed and the rest 5 measurements were averaged and expressed in the form of (mean ± Sd).

The lesions that failed to measure SWV were shown as X.XX m/s and were treated as 9 m/s. To evaluate the sensitivity, specificity, and accuracy of SWV in different size of breast lesions, the lesions were divided into three groups according to the size: Group 1: maximum diameter <10 mm; Group 2: maximum diameter between 10 and 20 mm; Group 3: maximum diameter >20 mm. The final diagnoses were confirmed by pathological examination after surgical removal, and the receiver-operating characteristic curve (ROC) analyses were performed in each group.

### 2.3. Statistical Analysis

SPSS 17.0 was applied for statistical analysis. Bivariate correlation analysis was performed to calculate the correlation coefficient. Difference in quantitative variables was compared with independent* t*-test, and qualitative variables were compared with *χ* ^2^ test. MedCalc 11 was used to make the receiver-operating characteristic curve (ROC) analyses and the area under curves (AUC) was compared using the* z* test. *P* < 0.05 was considered as statistical significance.

## 3. Results

Overall 146 women with 206 breast lesions were involved in the study, among which 33.5% (69/206) lesions were in the screening and 66.5% (137/233) lesions were with symptoms. Characteristics of patients and the lesions were listed in Table 1. The mean age of patient with malignant lesions was 58.8 ± 11.2 (range: 31–91 years), which is much older than patients with the benign lesions (mean: 40.8 ± 11.1 years, range: 19–74 years, *P* < 0.01). Significant difference was also found in boundary, echogenicity, microcalcification, and aspect ratio. Generally, the benign lesion always showed a clear boundary, iso- or hypoechogenicity, and the aspect ratio of the majority of benign lesions tends to be <1. As the microcalcification display of ultrasound was not as sensitive as mammography, most lesions showed nonmicrocalcification. However, the proportion of microcalcification in malignant lesion was still higher than that in benign ones.

Totally 163 lesions were diagnosed as benign, including 106 cases of fibroadenoma, 49 cases of mastopathy, 5 cases of intraductal papilloma, 2 cases of lipoma, and a granulomatosis. Forty-three lesions were diagnosed as malignant, including 25 cases of invasive ductal carcinoma, 9 cases of ductal papilloma (DCIS), 6 cases of mucinous carcinoma, 2 cases of invasive-lobular carcinoma, and a diffuse large B-cell lymphoma. In total, 16 lesions failed to measure SWV and showed X.XX m/s, including 9 invasive ductal carcinomas, 3 DCIS, 2 mucinous carcinomas, 1 fibroadenoma with calcification, and 1 mastopathy with calcification. The pathological results of the false positive lesions were shown in two cases with calcification. The SWV of the two benign lesions was much higher than that of typical benign lesion, which indicated that the value of SWV could be influenced by the pathological types.

A comparison of SWV between the benign and malignant lesions was shown in Table 2. To the whole lesions involved, SWV of the malignant lesions was (6.17 ± 2.58 m/s, range: 1.13–9 m/s) m/s, which was significantly higher than that of benign lesions (2.36 ± 1.21 m/s, range: 0.74–9 m/s, *P* < 0.001). Meanwhile, mean SWV of the benign and malignant lesions in each group was also shown in this table. In Group 2 (10–20 mm, *n* = 101) and Group 3 (>20 mm, *n* = 37), SWV of malignant lesions was much higher than that of the benign (*P* < 0.001), whereas the difference was not obvious in Group 1 (<10 mm, *n* = 68, *P* = 0.15), which indicated the difference of SWV was not obvious to lesion <10 mm. Besides, there was no statistical difference of SWV in normal breast tissue adjacent to the breast lesions, neither for the whole lesions (*P* = 0.62), nor between each groups (*P* = 0.56, *P* = 0.92, and *P* = 0.80, resp.).

**Figure 3:**
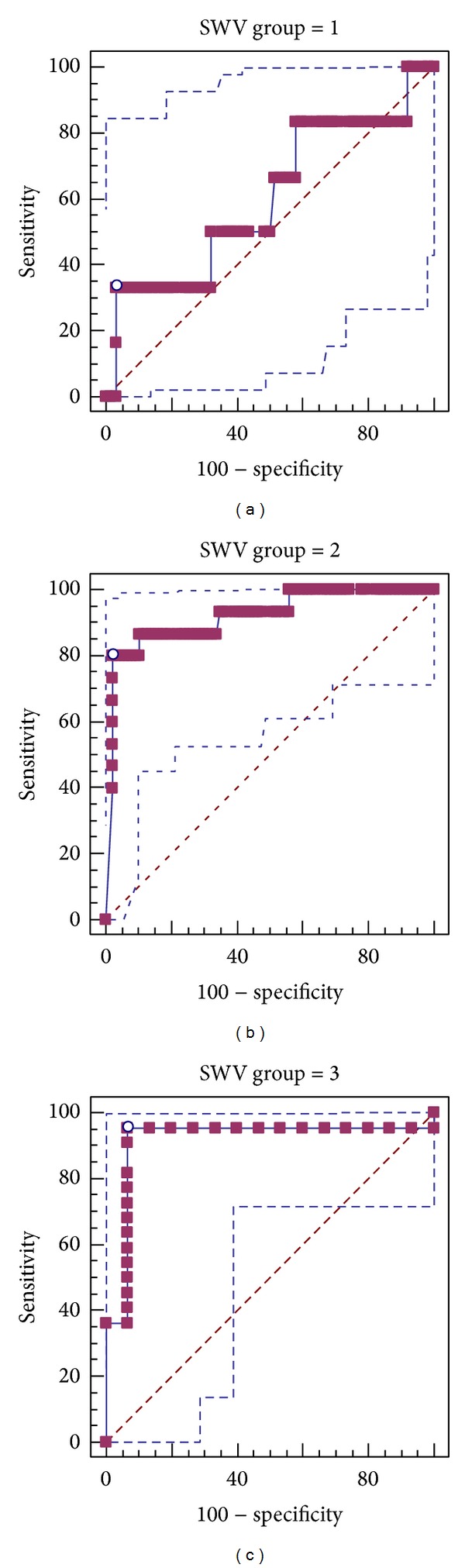
The ROC of three groups was compared with* z* test; (a) lesion < 10 mm, (b) lesions between 10 and 20 mm, and (c) lesions over 20 mm.

The best cut-off value of SWV for diagnosis of malignant breast lesion was 4.22 m/s, and based on this, sensitivity, specificity, and accuracy of SWV in different groups were presented in Table 3. The sensitivity, specificity, and accuracy for SWV to all lesions were 81.4.00%, 96.32%, and 91.21%, respectively, and the AUC was 0.886 (95% CI: 0.834–0.926, *P* < 0.0001). The AUC of three groups was 0.601 (95% CI: 0.475–0.718), 0.919 (95% CI: 0.848–0.964), and 0.915 (95% CI: 0.776–0.981), for the Groups 1, 2, and 3, respectively (Figure 3). There was significant difference between Group 1 (<10 mm, *n* = 68) and Group 2 (10–20 mm, *n* = 101, *P* = 0.0343 < 0.05), as well as between Group 1 (<10 mm, *n* = 68) and Group 3 (>20 mm, *n* = 37, *P* = 0.0437 < 0.05). Sensitivities of three groups were 33.33%, 80%, and 95.45%, respectively, and there was an escalating tendency of sensitivity along with the increasing lesion size.

**Figure 4:**
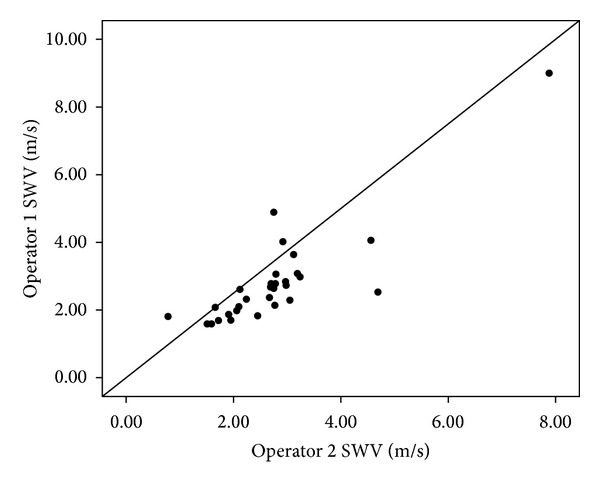
Another subgroup of 30 lesions was selected to investigate interobserver reproducibility of two independent operators, and the correlation coefficient was 0.857 (*P* < 0.01).

The mean SWV of the two independent operators is shown in Figure 4, and the correlation coefficient was 0.857 (*P* < 0.01). There is no significant difference in age, lesion size, pathological results, or initial examination of reasons (screening or having symptoms).

## 4. Discussion

In recent year, the incidence and prevalence of breast cancer increase at a high rate, and the detection of breast lesions is particularly essential to improve life expectancy [11, 12]. The quantitative diagnosis of small breast lesion is difficult in clinical practice. Despite palpation, lesion profile between the benign and the malignant is not obvious at the early stage [13]. As a noninvasive technology, VTTQ of ARFI showed excellent performance in differentiating the breast lesion. However, the results of our study demonstrated that the sensitivity of VTTQ in lesions <10 mm was relatively low (33.33%). Previous studies form Yoon et al. [14] evaluated the discordant of the elastography pathology and indicated that the elastography was unsuitable in the diagnosis of lesions <10 mm or >20 mm, because of that it is less clear to interpret the information in small breast lesion with the small ROI, and it is harder to perform homogeneous compression in larger breast lesions. Our study is partly consistent with their conclusion, and the sensitivity of VTTQ was relatively low for small lesion might lie on the fact that the two-dimensional image of ultrasonography was a single plane of the lesion, but the lesion was a spatial structure. When the ROI was put in the middle of small lesion, it is more likely to include normal breast tissue, which will result in deviation of the SWV. In addition, examination of small breast lesions is much more likely to be affected by respiratory movement, which would also influence the value of SWV. Taking together the above reasons, the sensitivity of VTTQ for lesions <10 mm was not satisfying. However, it is noteworthy that the specificity of lesion <10 mm is relatively high (96.77%), which indicates that, for a small lesion with a high SWV value, it is more likely to be malignant. As for the lesions >20 mm, we already exclude the obvious cystic lesion, so sensitivity, specificity, and accuracy were basically acceptable.

Conventional ultrasonography was mainly based on morphological changes, such as boundary, echogenicity, and microcalcification [15]. A typical benign lesion, such as fibroadenoma, usually presents clear boundary, low echogenicity, and expansive growth pattern [16]. On the contrary, the malignant lesions usually occur in elderly women, with an irregular boundary, an infiltrative growth of which the D/W ratio >1. Besides, microcalcification was particularly often seen in the malignant lesion [17]. However, the conventional ultrasound is not sensitive for microcalcification, and there is a remarkable overlap between the benign and malignant lesions, which is a problem remaining in routine clinical practice. Novel technique like VTTQ of ARFI may overcome those shortages and provides objective assessment for breast lesion. There is now a general consensus that the stiffness was closely related to the properties of breast lesion [18, 19]. As the inherent properties, the elastic values from high to low were invasive ductal breast carcinoma, noninvasive ductal breast carcinoma, fibroadenoma, breast tissue, and fat tissue ordered by pathological types [20, 21]. The intercellular substance of benign breast lesions such as fibroadenoma was composed of mucopolysaccharides; thus, the tissue tends to be loose and soft, whereas the intercellular substance of malignant lesion such as invasive ductal carcinoma often is filled with dense fibrous tissue, which is much harder [22]. In our study, SWV of benign lesion is predominantly lower than malignant lesion (Figure 2), which is consistent with the previous studies.

There are several explanations for X.XX m/s. Previous studies have indicated X.XX was caused by lack of generation of shear waves or high shear wave attenuation, which meant poor signal-to-noise ratio [23, 24]. Studies from Wojcinski [9] indicated that the best accuracy would be achieved when X.XX m/s was set as the cut-off to differentiate malignant lesions from benign. In our study, X.XX was treated as 9 m/s, which is consistent with previous reports from Zhang et al. [8]. The replacement of X.XX m/s with SWV beyond 9.0 m/s is only a rough estimation of the actual SWV, and the exact value of SWV might be higher than the cut-off we obtain.

It has been reported that the observer variability was a major limitation of elastography due to the various degrees of the repetitive compression motions [25]. In our present study, the correlation coefficient is high (0.857, *P* < 0.01), which indicated the reproducibility of VTTQ is satisfactory.

The first generation of elastography obtained the elastic value by comparing the lesion with surrounding tissue, which is affected by the experience and skill of the operator [26]. On the contrary, VTTQ of ARFI obtained the elastic value by measuring SWV directly [27], which is more direct and objective [10]. But there is still some limitation of VTTQ. Firstly, as a new technology to diagnose breast lesion, VTTQ of ARFI cannot be completely separated from conventional ultrasound, and sensitivity of VTTQ for lesion <10 mm needs to be improved. Secondly, there is still some misdiagnosis of ARFI, and the reason might be related to the pathological types of lesion. When there is calcification, sclerosing lesion, or hyalinization that happened in benign lesions, SWV tends to be higher than typical benign lesion. Finally, as external pressure could also bias the results, and it could not be standardized at present [23], the VTTQ of ARFI may be less objective as we think. However, the VTTQ of ARFI is still a strong complement to conventional ultrasound, which could provide useful complement information about lesion qualitative diagnosis.

In summary, the VTTQ of ARFI is a promising method in the differential diagnosis of the malignant breast lesion, especially suited for breast lesions >10 mm. Further studies were warranted to improve the sensitivity of VTTQ assessment in breast lesion <10 mm.

## Figures and Tables

**Table 1:** Characters of breast lesions in transitional ultrasound.

	Benign	Malignant	*P*
Patients			
Mean age	40.8 ± 11.1	58.8 ± 11.2	<0.01
Median age	40	57	/
Boundary			<0.01
Clear	142	12	
Unclear	21	31	
Echogenicity			<0.05
Hyperechoic	14	5	
Iso-echoic	76	11	
Hypoechoic	73	27	
Calcification			<0.01
None	139	29	
Microcalcification	2	10	
Macrocalcification	22	4	
Depth width ratio			<0.01
<1	127	23	
>1	36	20	

**Table 2:** Comparison of shear wave velocities in the benign and malignant lesions.

	Group	Malignant	Benign	*P*
Lesion SWV (m/s)	1	2.96 ± 1.54	2.28 ± 1.03	0.15
	2	6.45 ± 2.59	2.35 ± 1.26	<0.001
	3	6.86 ± 2.18	2.70 ± 1.58	<0.001
	Total	**6.17 ± 2.58**	**2.36 ± 1.21**	**<0.001**

Normal breast tissue SWV (m/s)	1	1.80 ± 0.58	1.66 ± 0.55	0.56
	2	1.51 ± 0.72	1.53 ± 0.47	0.92
	3	1.68 ± 0.46	1.72 ± 0.42	0.80
	Total	**1.64 ± 0.57**	**1.60 ± 0.50**	**0.62**

**Table 3:** Performance of SWV in differentiating benign from malignant lesions in different size.

Groups	Sensitivity (%)	Specificity (%)	Accuracy (%)	AUC	95% CI	*z*	*P*
<10 mm	33.33 (2/6)	96.77 (60/62)	91.17 (62/68)	0.601	0.475–0.718	0.7	0.48
10–20 mm	80 (12/15)	96.51 (83/86)	94.06 (95/101)	0.919	0.848–0.964	9.76	<0.0001*
>20 mm	95.45 (21/22)	93.33 (14/15)	94.59 (35/37)	0.915	0.776–0.981	7.017	<0.0001**
Total	**81.40 (35/43)**	**96.32 (157/163)**	**91.21 (188/206)**	**0.886**	**0.834–0.926**	**10.245**	**<0.0001**

*Compared with the AUC of <10 mm, *z* = −2.116, *P* = 0.0343 < 0.05.

**Compared with the AUC of <10 mm, *z* = −2.017, *P* = 0.0437 < 0.05.
